# Detectability of Retinal Diffusion Restriction in Central Retinal Artery Occlusion is Linked to Inner Retinal Layer Thickness

**DOI:** 10.1007/s00062-022-01168-9

**Published:** 2022-05-03

**Authors:** E. Siebert, M. Rossel-Zemkouo, K. Villringer, K. Neumann, G. Bohner, L. A. Danyel

**Affiliations:** 1 Institute of Neuroradiology, Charité—Universitätsmedizin Berlin, corporate member of Freie Universität Berlin and Humboldt Universität zu Berlin, Berlin, Germany; 2 Department of Ophthalmology, Charité—Universitätsmedizin Berlin, corporate member of Freie Universität Berlin and Humboldt Universität zu Berlin, Berlin, Germany; 3 Center for Stroke Research Berlin, Charité—Universitätsmedizin Berlin, corporate member of Freie Universität Berlin and Humboldt Universität zu Berlin, Berlin, Germany; 4 Institute of Biometry and Clinical Epidemiology, Charité—Universitätsmedizin Berlin, corporate member of Freie Universität Berlin and Humboldt Universität zu Berlin, Berlin, Germany; 5 Department of Neurology, Charité—Universitätsmedizin Berlin, corporate member of Freie Universität Berlin and Humboldt Universität zu Berlin, Augustenburger Platz 1, 13353 Berlin, Germany

**Keywords:** Cerebrovascular disease, Stroke, Ischemia, Magnetic resonance imaging, Eye disease

## Abstract

**Purpose:**

To investigate retinal microstructure differences in central retinal artery occlusion (CRAO) patients with and without visible retinal diffusion restriction (RDR) on diffusion-weighted magnetic resonance imaging (DWI).

**Methods:**

Consecutive CRAO patients with available optical coherence tomography (OCT) and DWI, both performed within 7 days after symptom onset, were included in a retrospective cohort study. The OCT scans were reviewed to assess retinal layer thickness, optical intensity and structural integrity. The OCT findings were compared between patients with and without visible RDR on DWI using Mann-Whitney U or Pearson’s Χ^2^ test.

**Results:**

A total of 56 patients (mean age 70.8 ± 12.8 years) were included. RDR was observed in 38 subjects (67.9%) with visually correlating low ADC map in 26 of 38 cases (68.4%). Superior and inferior parafoveal macular thickness measurements (SMT, IMT) of RDR negative patients were significantly lower when compared to RDR+ patients (370.5 ± 43.8 µm vs. 418.2 ± 76.0 µm, *p* = 0.016; 374.4 ± 42.9 µm vs. 428.8 ± 63.2 µm, *p* = 0.004) due to differences in inner retinal layer thickness (IRLT, 188.8 ± 34.4 µm vs. 234.7 ± 49.0 µm, *p* = 0.002). IRLT values of RDR negative patients were higher in 1.5T compared to 3T the DWI (205.0 ± 26.0 µm vs. 168.6 ± 32.8 µm, *p* = 0.026).

**Conclusions:**

Detectability of RDR is likely contingent upon the degree of ischemic retinal swelling in CRAO. Technical adjustments to the DWI protocol, such as increased field strength, may improve visibility of RDR.

**Supplementary Information:**

The online version of this article (10.1007/s00062-022-01168-9) contains supplementary material, which is available to authorized users.

## Introduction

Retinal diffusion restriction (RDR) has recently been identified as a regular finding on diffusion-weighted magnetic resonance imaging (DWI-MRI) in patients with central retinal artery occlusion (CRAO) [1–5]. With increasing utility and availability of emergency MRI in stroke [6, 7], RDR might contribute to the diagnosis of retinal ischemia, streamlining the integration of CRAO patients into existing vascular emergency networks and possibly improving the access to recanalization therapy; however, sensitivity of RDR in CRAO as reported for standard 1.5T and 3T brain stroke DWI sequences has been limited (62.6–75.0%) [2, 5]. On the one hand, detectability of RDR on DWI is likely to depend on the time delay between symptom onset and DWI, as RDR were reported to be less frequent in DWI performed 1 week after onset of vision loss [5]. On the other hand, differences in the extent of retinal cell edema might influence the magnitude of diffusion restriction, thereby affecting the ability to produce a perceivable DWI signal increase in the affected voxels encompassing the inner retinal layers.

Optical coherence tomography (OCT) is an interferometric imaging technique that allows for the noninvasive examination of retinal layers with spatial resolutions in the micrometer range [8]. Several studies have demonstrated its utility in visualizing retinal microstructure changes due to ischemic damage in CRAO [9–15]. Intracellular edema with inner retinal layer swelling [9] and loss of ganglion layer transparency [9, 10] are among the characteristic features described secondary to breakdown of retinal arterial perfusion and developing ischemia. We designed a retrospective cohort study to investigate differences in retinal microstructure between CRAO patients with and without discernible RDR on DWI-MRI.

## Material and Methods

Study approval was obtained from the local ethics committee (EA1/177/19). All methods were performed in accordance with relevant guidelines. Written informed consent was obtained from all participants.

### Patients

This retrospective study included consecutive CRAO patients treated in our neurovascular department between January 2010 and 2019 with available brain diffusion-weighted imaging and optical coherence tomography, both performed within 7 days after symptom onset. All patients met diagnostic criteria of nonarteritic CRAO with sudden onset of painless, monocular visual impairment and presence of characteristic fundoscopic features (cherry-red spot sign or retinal opacity, visible emboli, attenuated retinal arterioles and/or optic disc pallor/edema). Patients with amaurosis fugax, branch retinal arteriolar occlusion or patients with symptoms suggestive of giant cell arteritis (according to the American College of Rheumatology 1990 criteria) were not considered for this study.

### Diffusion-weighted MR Imaging

Magnetic resonance imaging was routinely performed at our institution to detect concurrent ischemic stroke and assess disease etiology in CRAO patients. EPI-DWI sequences were acquired on 1.5T (Aera, Siemens, Erlangen, Germany) and 3T scanners (Skyra, Siemens) with 20 channel head coils. On another 3T scanner (Trio, Siemens) DTI sequences were routinely acquired for DWI calculation. Slice thicknesses (ST) were as follows: 2.5 mm (DTI), 3 mm and 7 mm (DWI). Trace DWI b = 1000 s/mm^2^ images from EPI-DWI sequences or calculated from EPI-DTI sequences were assessed by a board-certified neuroradiologist (> 15 years of experience) blinded for clinical patient data (e.g. side of the affected eye) and OCT scan assessments. Presence of RDR on DWI was noted, if an abnormal signal increase of the inner wall of the globe was present on at least two adjacent slices, when compared to the contralateral eye. Additionally, ADC maps were evaluated for the presence of visually correlating low signal.

### Optical Coherence Tomography

Spectral domain OCT scans were acquired using a commercially available device (Spectralis ®, Heidelberg Engineering GmbH, Heidelberg, Germany) and its software (Heidelberg Eye Explorer 1.9.14.0 with Spectralis Viewing Module Calculator Data Provider/Manager 1.0.11.0, Heidelberg Engineering). A 5.8 mm × 4.4 mm protocol with acquisition of 19 horizontal B‑scans was employed to obtain a volume scan of the macular region of the affected and unaffected eye. All OCT scan assessments were performed by a board-certified ophthalmologist (> 10 years of experience).

**Fig. 1:**
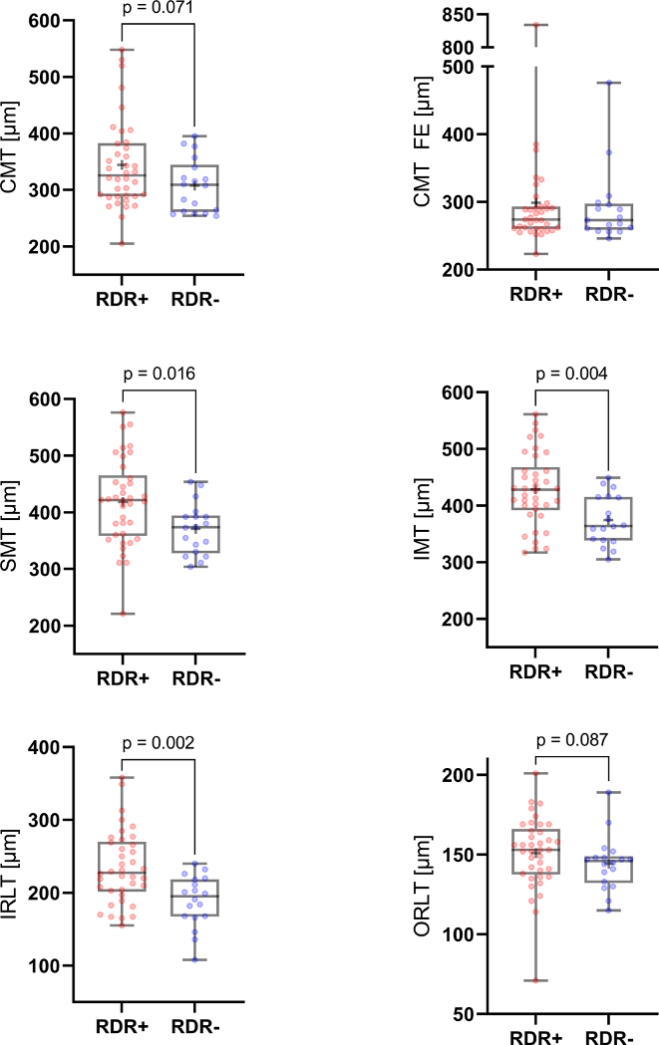
Differences in retinal layer thickness measurements between CRAO patients with (RDR+) and without visible (RDR−) restricted retinal diffusion on standard diffusion-weighted magnetic resonance imaging (DWI). OCT-based parafoveal macular thickness measurements (SMT, IMT) and IRLT of CRAO patients with no detectable RDR on DWI were significantly lower when compared to RDR+ patients. No significant differences were observed for central (foveal) macular thickness of the affected or the fellow eye (FE) and ORLT. *CMT* central macular thickness, *CRAO* central retinal artery occlusion, *IMT* inferior parafoveal macular thickness, *IRLT* inner retinal layer thickness, *OCT* optical coherence tomography, *ORLT* outer retinal layer thickness, *RDR* retinal diffusion restriction, *SMT* superior parafoveal macular thickness

All macular volume scans were checked for foveolar centration of the Early Treatment Diabetic Retinopathy Study (ETDRS) grid and layer segmentation and corrected manually in cases of decentration or segmentation errors. Central macular thickness (CMT), which represents the average thickness of the foveal 1 mm diameter-zone, was obtained for the affected and fellow eye. Superior (SMT) and inferior (IMT) parafoveal macular thicknesses were measured within the superior and inferior sector of the 3 mm diameter zone of the ETDRS grid of the affected eye. Inner (IRLT) and outer (ORLT) parafoveal retinal layer thicknesses were measured manually, 1000 µm temporal from the foveola of the affected eye with the virtual ruler tool in a perpendicular direction to the retinal pigment epithelium (RPE). Based on the findings and methods of previous studies [16–18], all layers between the inner limiting membrane (ILM) and the outer border of the outer plexiform layer (OPL) were defined as the inner retinal layers. The outer nuclear layer (ONL), external limiting membrane (ELM) and the photoreceptor layer (PR) were subsumed as the outer layers of the neurosensory retina. The analysis protocol for retinal layer thickness measurements is illustrated in the data supplement (Fig. 1, Supplementary Material).

**Fig. 2:**
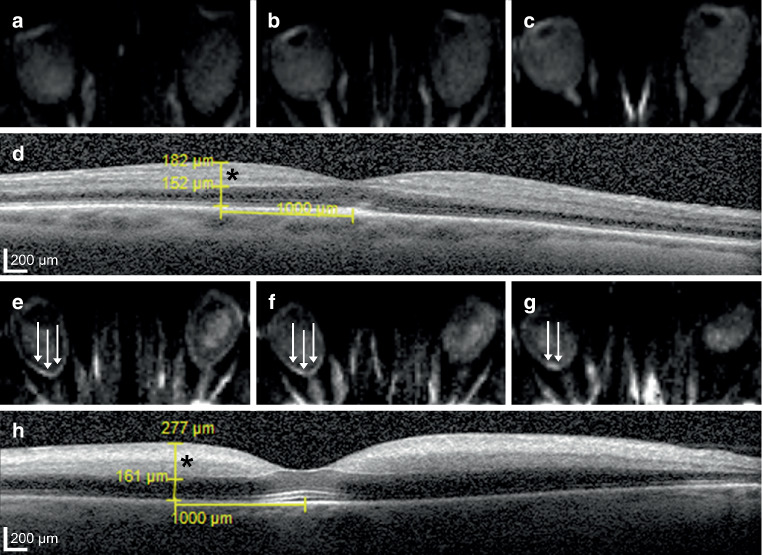
Representative examples of IRLT measurements in CRAO patients with and without visible restricted retinal diffusion on 3T DWI-MRI (2.5 mm calculated DWI-TRACE from DTI-EPI). **a**, **b**, **c** No demonstrable restricted diffusion is visible on retinal DWI of a 70-year-old woman with right-sided CRAO. **d** The corresponding SD-OCT cross-section shows mild swelling of the inner retinal layer (182 µm, *black asterisk*). **e**, **f**, **g** A marked retinal DWI hypersignal is noted on three adjacent slices in a 67-year-old man with right-sided CRAO (*white arrows*). **h** The corresponding SD-OCT image reveals severe inner retinal layer edema (277 µm, *black asterisk*). *CRAO* central retinal artery occlusion, *DWI-MRI* diffusion-weighted magnetic resonance imaging, *IRLT* inner retinal layer thickness, *SD-OCT* spectral-domain optical coherence tomography, *TRACE* , *EPI*

**Fig. 3:**
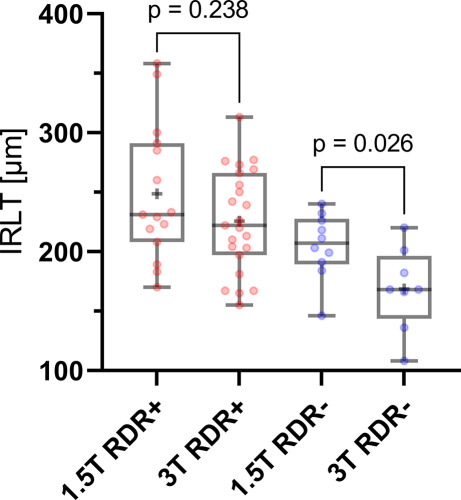
Comparison of inner retinal layer thickness (IRLT) measurements in CRAO patients with (RDR+) and without visible (RDR−) restricted retinal diffusion on 1.5 and 3T standard DWI-MRI. Scattered boxplots indicate improved detectability of RDR on 3T, when compared to 1.5T DWI-MRI in ischemic IRL edema with corresponding IRLT measurements between 200 µm and 250 µm. Accordingly, IRLT of RDR− patients on 3T DWI-MRI was lower, when compared to RDR− patients on 1.5T DWI-MRI. *CRAO* central retinal artery occlusion; *DWI-MRI* diffusion-weighted magnetic resonance imaging; *RDR* retinal diffusion restriction

The presence of a prominent middle limiting membrane (*p*-MLM) sign was noted, if a hyperreflective line at the level of the OPL was present on any of the 19 B-scans. Structural loss of the inner retinal layers was graded as follows: partial loss of retinal layer structure (grade I), severe but incomplete loss of retinal layer structure (grade II) or complete loss of inner retinal structure (grade III). Similarly, IRL hyperreflectivity was graded on a semi-quantitative scale (grades I–III). Additionally, the presence of ORL hyporeflectivity was noted. Visual examples of different grading in IRL hyperreflectivity and retinal structure loss are provided in the data supplement (Fig. 2 and Fig. 3, Supplementary Material).

### Statistical Analysis

GraphPad Prism (GraphPad Prism version 8.0.0 for Windows, GraphPad Software, San Diego, CA, USA) and IBM SPSS Statistics software (IBM SPSS Statistics for Windows, Version 25.0. Armonk, NY, USA) were employed for statistical analysis. We compared retinal layer thickness measurements (CMT, SMT, IMT, IRLT, ORLT) and the presence of varying OCT features in CRAO (*p*-MLM sign, loss of retinal layer structure, IRL hyperreflectivity or ORL hyporeflectivity) between patients with and without detectable RDR on DWI using Mann-Whitney *U*- or χ^2^-tests, respectively. In this observational study no correction for multiple testing was performed. All reported *p*-values are exploratory. A *p*-value *p* was deemed significant if *p* ≤ 0.05. Descriptive statistics are presented as mean ± SD.

## Results

### Patient Cohort

After exclusion of 4 subjects due to DWI artifacts and 1 subject due to severe age-related macular degeneration, 56 CRAO patients (mean age 70.8 ± 12.8 years; 30 female) with available DWI and OCT were included in this study. Detailed information on clinical characteristics of CRAO patients, including cardiovascular risk profiles and fundoscopic features are given in the data supplement (see Table 1, Supplementary Material). Side distribution of retinal ischemia was balanced with 29 (51.8%) right-sided and 27 (48.2%) left-sided occlusions. Mean visual acuity on initial presentation was 1.82 logMAR (43/56 patients or 76.8%; calculation according to Schulze-Bonsel et al. [19]). Of the patients 13 (23.2%) had visual acuity of light perception or worse, which could not be quantified on a comparable scale.

### Diffusion-weighted Imaging Analysis

A total of 56 MRI scans (field strength 1.5T in 25 or 44.6% and 3T in 31 or 55.4% of cases) were assessed. The sequence types employed were as follows: 2.5 mm calculated DWI-TRACE from DTI-EPI (23 or 41.1%), 3 mm DWI-EPI TRACE (31 or 55.4%) and 7 mm DWI-EPI TRACE (2 or 3.6%). Time intervals between clinical onset of visual impairment and MRI were > 12–24 h in 3 (5.4%), > 24–72 h in 37 (66.1%) and > 72 h–7 days in 14 (25.0%) cases. An unambiguous assignment to a time group was not possible in 2 patients (3.6%). The mean time interval between DWI and OCT was 32.6 ± 23.3 h. Retinal diffusion restrictions (RDR) were observed in 38 patients (67.9%; 23/31 or 74.2% in 3T and 15/25 or 60.0% in 1.5T scans) with visually correlating low signal on the ADC map in 26 cases (68.4% of RDR− positive scans). Multiple comparison χ^2^-test revealed no differences in the presence of RDR between time groups (*p* = 0.1).

### Optical Coherence Tomography Analysis

Time intervals between clinical onset of visual impairment and OCT examination were as follows: ≤ 4.5 h in 4 (7.1%), > 4.5–12 h in 7 (12.5%), > 12–24 h in 7 (12.5%), > 24–72 h in 20 (35.7%) and > 72 h–7 days in 14 (25.0%). An unambiguous assignment to a time group was not possible in 4 cases (7.1%). Central foveal macular thickness (CMT) among CRAO patients was 332.6 ± 71.2 µm (median 319.0; Q1 282.5; Q3 365.8). Superior (SMT) and inferior (IMT) parafoveal macular thickness were 402.9 ± 70.9 µm (median 392.0; Q1 351.0; Q3 445.8) and 411.3 ± 62.8 µm (median 411.5; Q1 359.0; Q3 445.3), respectively. In comparison, CMT of the fellow eye was 296.5 ± 86.1 µm (median 273.5; Q1 260.8; Q3 293.8). Inner (IRLT) and outer (ORLT) parafoveal retinal layer thickness of the affected eye were 220.0 ± 49.7 µm (median 218.5; Q1 183.8; Q3 244.0) and 148.9 ± 21.0 µm (median 148.0; Q1 137.5; Q3 159.5). A prominent middle limiting membrane (*p*-MLM) sign was present in 50 (89.3%) cases. Increased IRL hyperreflectivity was observed in 52 patients (92.9%) with grade I in 19 (36.5%), grade II in 16 (30.8%) and grade III in 17 (32.7%) cases. Hyporeflective outer retinal layers were noted in 44 (78.6%) patients. Loss of retinal layer structure was visible in 40 (71.4%) cases and graded as follows: grade I 15 (37.5%), grade II 9 (22.5%) and grade III 16 (40.0%).

### Comparison of OCT Findings Between DWI Positive and Negative Patients

Table 1 and Fig. 1 detail retinal layer thickness measurements in CRAO patients with and without visible restricted retinal diffusion on diffusion-weighted magnetic resonance imaging. Parafoveal macular thickness measurements (SMT, IMT) of CRAO patients with no detectable RDR on DWI were significantly lower when compared to RDR+ patients (SMT: 370.5 ± 43.8 µm vs. 418.2 ± 76.0 µm, *p* = 0.016; IMT: 374.4 ± 42.9 µm vs. 428.8 ± 63.2 µm, *p* = 0.004). Central (foveal) macular thickness. however, did not differ significantly between the two groups (307.6 ± 45.8 µm vs. 344.4 ± 77.7 µm, *p* = 0.071). The observed differences in parafoveal macular thickness could be attributed to differences in IRLT, which were significantly lower in CRAO patients without visible RDR on DWI (188.8 ± 34.4 µm vs. 234.7 ± 49.0 µm, *p* = 0.002). ORLT measurements did not vary significantly (*p* = 0.087). Fig. 2 illustrates representative examples of IRLT measurements in CRAO patients with and without visible restricted retinal diffusion on 3T DWI-MRI.

**Table 1:** OCT-based retinal layer thickness measurements in CRAO patients with (RDR+) and without visible (RDR−) restricted retinal diffusion on standard diffusion-weighted magnetic resonance imaging

	RDR+ (*n* = 38)					*RDR− (n* *=* *18)*					*p*-value
	*M*	*SD*	*Mdn*	*Q1*	*Q3*	*M*	*SD*	*Mdn*	*Q1*	*Q3*	
CMT (µm)	344.4	77.7	325.5	289.3	380.0	307.6	45.8	309.0	262.5	335.3	0.071
CMT FE (µm)	298.9	97.7	274.0	260.5	292.0	291.4	54.5	273.0	261.0	296.0	0.938
SMT (µm)	418.2	76.0	421.5	360.5	458.3	370.5	43.8	373.5	333.3	392.0	0.016
IMT (µm)	428.8	63.2	428.0	395.5	461.0	374.4	42.9	364.0	339.5	414.8	0.004
IRLT (µm)	234.7	49.0	227.5	203.3	268.3	188.8	34.4	195.0	168.0	216.3	0.002
ORLT (µm)	151.1	22.5	153.0	138.5	164.8	144.4	16.4	146.0	134.5	148.0	0.087

*CMT* central macular thickness, *CMT FE* central macular thickness fellow eye, *CRAO* central retinal artery occlusion, *IMT* inferior parafoveal macular thickness, *IRLT* inner retinal layer thickness, *OCT* optical coherence tomography, *ORLT* outer retinal layer thickness, *RDR* retinal diffusion restriction, *SMT* superior parafoveal macular thickness

Multiple comparison χ^2^-testing identified increased frequency of grade III IRL hyperreflectivity (Fig. 4 of Supplementary Material; 0/18 or 0% RDR− vs. 17/38 or 44.7% RDR+ with *p* = 0.0007) and complete loss of inner retinal layer structure (grade III; Fig. 4 of Supplementary Material; 1/18 or 5.6% RDR− vs. 15/38 or 39.5% RDR+ with *p* = 0.009) in patients with visible RDR. No differences were observed for the presence of *p*-MLM sign and hyporeflectivity of the ORL (18/18 or 100.0% RDR− vs. 32/38 or 84.2% RDR+, *p* = 0.074 and 13/18 or 72.2% vs. 31/38 or 81.6%, *p* = 0.425).

### Comparison of IRLT and Presence of RDR in 1.5T and 3T DWI-MRI

Figure 3 compares IRLT measurements in patients with and without RDR on 1.5 and 3T DWI-MRI. While IRLT measurements did not differ between RDR+ patients on 1.5T and 3T DWI (248.5 ± 55.4 µm vs. 225.7 ± 41.9 µm, *p* = 0.238), IRLT was higher in RDR− patients on 1.5T, when compared to 3T MRI (205.0 ± 26.0 µm vs. 168.6 ± 32.8 µm, *p* = 0.026).

## Discussion

Since the first description in four patients with retinal ischemia and concurrent carotid arteriopathy [3, 4], RDR has been identified as a characteristic finding on DWI-MRI in patients with acute CRAO and branch retinal arteriolar occlusion [2, 5, 20]. As such, there has been increasing interest in the utility of retinal DWI as a supplemental tool for the diagnosis of retinal ischemia, e.g. in instances of obstructed visibility of the fundus; however, sensitivity of RDR in CRAO as reported in previous studies utilizing standard stroke DWI has been limited (62.6–75.0%) [2, 5]. Embolic occlusion of the central retinal artery causes abrupt cessation of blood flow to the inner retinal layers with subsequent development of cytotoxic edema and retinal necrosis [21]; however, relevant differences in the severity of structural retinal changes have been reported for CRAO patients using OCT [9, 22].

Our study substantiates that visibility of RDR on DWI-MRI is contingent upon the degree of ischemic retinal swelling, which provides a plausible explanation for the limited sensitivity of RDR in CRAO reported previously [2, 5]. Accordingly, we found significantly lower IRLT in DWI negative CRAO patients, when compared to patients with visible RDR. No differences were found for ORLT measurements, since the outer retinal layers, including the photoreceptor cells, are supplied by the posterior ciliary system and not primarily affected by CRAO. It is reasonable to infer that in patients with mild cytotoxic edema of the inner retinal layers no perceivable signal increase is produced on standard DWI-MRI. Similarly, parafoveal macular thickness measurements (SMT, IMT) were significantly lower in RDR− patients; however, no statistically relevant difference was observed for foveal macular thickness measurements (CMT). These observations can be explained by the retinal distribution of ganglion cells, which increase in number towards the center of the macula but are progressively reduced from the fovea to the foveolar region [23]. Since the ganglion cell layer is mainly affected by retinal ischemia in CRAO, parafoveal macular layers show pronounced swelling, while edema of the fovea remains discreet, resulting in the characteristic fundoscopic feature of cherry-red-spot sign in CRAO [24]. Interestingly, many CRAO patients with visible RDR on DWI show slight retinal thickening corresponding to the central macular region of the posterior globe [2].

Danyel et al. reported higher detection rates of RDR in CRAO for 3T, when compared to 1.5T DWI-MRI (72.2% vs. 59.6%), although this difference did not meet statistical significance (*p* = 0.135) [5]. Similarly, our data indicate that higher field strength improves detection of weak RDR in mild retinal edema, as IRLT measurements were significantly higher in 1.5T DWI of RDR− patients when compared to 3T DWI.

Two recent studies investigated the time-dependency of retinal edema in acute CRAO using OCT [11, 25]. The authors found that retinal ischemia caused a relative retinal thickness increase (RRTI) following a hyperbolic curve with nearly linear progression within the first 10 h after CRAO onset. Consequently, IRL edema is not fully developed in patients presenting within the proposed 4.5 h window for thrombolytic therapy. Considering these findings it is likely that detection of RDR in the early stages of CRAO is challenging, because only a weak signal increase is produced on DWI-MRI. On the other hand, visibility of RDR on DWI-MRI may indicate an advanced stage of retinal ischemia in patients with unknown time of onset. This is of relevance, because as much as one third of CRAO patients discover visual impairment on waking up in the morning [26], which precludes the assessment of retinal ischemia duration. Prospective trials are needed to explore DWI signal evolution and optimize DWI-MRI protocols for orbital imaging in acute CRAO. Novel DWI sequence techniques, such as fast spin echo radial acquisition DWI, small FOV DWI and readout-segmented DWI sequences might help to improve signal-to-noise ratio, as well as reduce distortion and susceptibility artifacts. Spectral-domain OCT could provide a valuable reference method for assessing the utility of different DWI sequences in the detection of RDR, as it provides quantification of IRL swelling.

Our study has several limitations, mainly inherent to its retrospective design, time delay between OCT and DWI-MRI and moderate overall case number, which have to be taken into account when interpreting its results. We used routine brain MRI protocols, not optimized for the visualization of the retina and DWI acquisition overall was heterogeneous, as different scanners, receive coils and sequences were employed. Consequently, variations in spatial resolution and signal-to-noise ratio may affect RDR detection. As stated before, we did not perform ADC value measurements due to significant partial volume averaging effects [2]. Further limitations are the absence of a control cohort and the single reader employed; however, interrater and intrarater reliability as well as test quality criteria of RDR in standard DWI have already been evaluated in previous studies: The assessment of RDR was accomplished with high specificity (0.80–1.00) and negative predictive value (0.76–0.80) in a CRAO study cohort with stroke controls [2]. Additionally, considerable interrater (κ = 0.70) and intrarater agreement (κ = 0.75) were reported for the identification of RDR in CRAO [2, 5]. Recent studies suggested a potential benefit of recombinant tissue plasminogen activator in CRAO, when administered within 4.5 h of symptom onset [27], a hypothesis that is currently being investigated by two multicenter RCTs (THEIA NCT03197194 and REVISON NCT04965038); however, our study did not include patients with DWI performed within the proposed 4.5‑h window for thrombolytic therapy, due to the significant time delay with which CRAO patients usually present to the neurovascular center. Similarly, MacGrory et al. reported a median delay of 8 h between symptom onset and admission to the emergency department [27]. It is important to note, however, that the initial contact to any medical provider reported for this cohort was distinctly earlier (median delay of 4 h). A reliable retinal DWI protocol for the diagnosis of acute CRAO would provide an opportunity to directly integrate retinal ischemia patients into existing neurovascular emergency networks, which would reduce time to thrombolytic treatment. A prospective trial investigating early DWI in CRAO is currently recruiting patients at our institution.

To conclude, our study provides evidence that detectability of RDR on DWI-MRI in CRAO patients is dependent on the degree of ischemic retinal swelling. Hence, severity of cytotoxic edema, as assessed by OCT, determines the extent of signal increase on DWI. This observation likely explains the limited sensitivity of RDR in CRAO reported in previous investigations. Technical adjustments of the DWI protocol, such as increased field strength may help to improve visibility of RDR. Further studies regarding the clinical utility of RDR for the estimation of ischemia duration in CRAO with unknown time of onset or its application for therapeutic decisions are warranted.

## Supplementary Information

Supplemental clinical information on CRAO patients, illustrated methodological information on OCT analysis and results of IRL hyperreflectivity and retinal structure loss analysis.
