# Supplementary material for: Detectability of Retinal Diffusion Restriction in Central Retinal Artery Occlusion is Linked to Inner Retinal Layer Thickness
Source: Clin Neuroradiol. 2022 May 3;32(4):1037–44. doi: 10.1007/s00062-022-01168-9 (PMC9744698; doi:10.1007/s00062-022-01168-9)
Supplement: Supplementary file 1 — Supplemental clinical information on CRAO patients, illustrated methodological information on OCT analysis and results of IRL hyperreflectivity and retinal structure loss analysis. [file 62_2022_1168_MOESM1_ESM.docx]

**Supplementary Material**

**Supplementary patient data**

| Patient characteristics | n (56) | % |
| --- | --- | --- |
| Cerebrovascular risk factors | | |
| Acute cerebral infarction | 15 | 26.79% |
| Atrial fibrillation | 5 | 8.93% |
| Carotid artery stenosis | 16 | 28.57% |
| Diabetes mellitus type 2 | 15 | 26.79% |
| Dyslipidemia | 40 | 71.43% |
| Hypertension | 41 | 73.21% |
| Smoking | 15 | 26.79% |
| RT-PA treatment | 11 | 19.64% |
| Fundoscopic features | | |
| Attenuated arteries | 31 | 55.36% |
| CRSS/retinal opacity | 49 | 87.50% |
| Optic disc pallor/edema | 25 | 44.64% |
| Visible emboli | 12 | 21.43% |

**Table 1:** **Cerebrovascular risk profile, frequency of thrombolytic treatment and fundoscopic findings in patients with central retinal artery occlusion.** CRSS, cherry red spot sign; RT-PA, recombinant tissue plasminogen activator.

**Supplementary technical data on MRI**

As recently described(1), diffusion-weighted MRI scans were acquired on 2 1.5T scanners (Aera, Siemens, Erlangen, Germany) with 20 channel head coils each and a 3T scanner (Skyra, Siemens, Erlangen, Germany) with a 20 channel head coil. The DTI sequence used for DWI calculation was acquired on a 3T scanner (Trio, Siemens, Erlangen, Germany) with a 32-channel head coil.

The DWI sequence parameters were: a) 3T: traced three-directional DWI EPI sequence, b-values 0 and 1000 s/mm2, slice thickness 3 mm, interslice gap 0.3 mm, number of averages 3, echo time 98, repetition time 10.3, number of phase encoding steps 143 , echo train length 71, percent sampling 100, percent phase field of view 100, pixel bandwith 1040, acquisition matrix 192, in-plane phase encoding direction AP, flip angle 90°, SAR 0.287, duration 2:36 min, b) 1,5T: traced three-directional DWI EPI sequence, b-values 0, 500 and 1000 s/mm2, slice thickness 3 mm, interslice gap 0.3 mm, number of averages 2, echo time 89, repetition time 8.80, number of phase encoding steps 105 , echo train length 53, percent sampling 80, percent phase field of view 100, pixel bandwith 1145, acquisition matrix 162, in-plane phase encoding direction AP, flip angle 90°, SAR 8.742, duration 2:22 min. c) 3T DTI EPI sequence, 6 directions, b-values 0 and 1000 s/mm2, traced DWI calculation, b-value 1000 s/mm2, slice thickness 2,5 mm, interslice gap 0 mm, number of averages 2, echo time 93, repetition time 8900, number of phase encoding steps 143 , echo train length 1, percent sampling 100, percent phase field of view 100, pixel bandwith 1240, acquisition matrix 192, in-plane phase encoding direction AP, flip angle 90°, SAR 0.282, duration 3:45 min.

**Supplemental Figures**

**
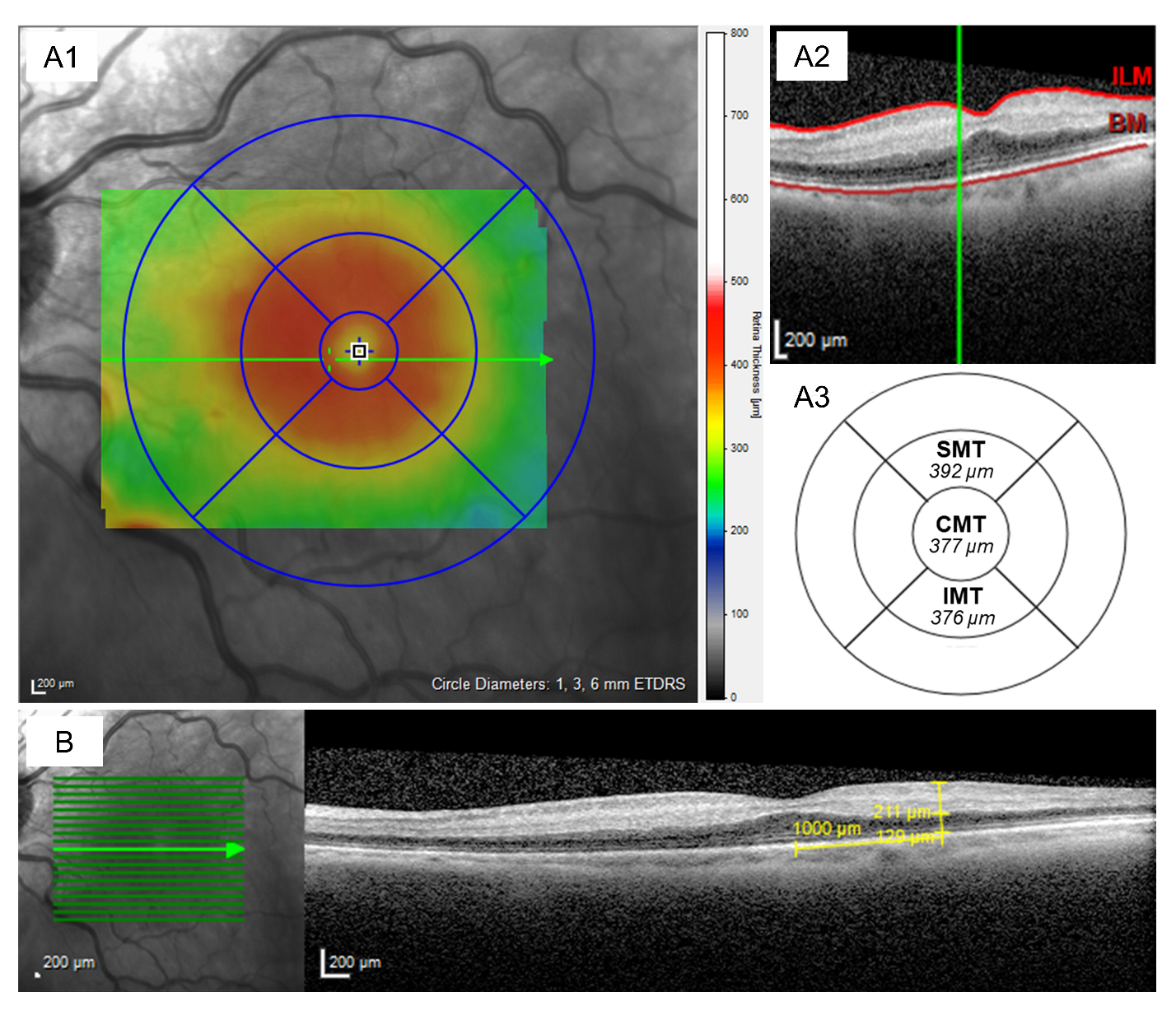
**

**Figure 1: Spectral-domain optical coherence tomography images illustrating the analysis protocol for retinal layer thickness measurements in patients with central retinal artery occlusion.** A1: Early Treatment Diabetic Retinopathy Study (ETDRS) grid with macular sectors superimposed on the macular region of the posterior pole of the eye(2). A2: Two-dimensional cross-section of the macular and foveal region. Macular thickness (SMT, CMT, IMT) was measured between the internal limiting membrane (ILM) and Bruch’s membrane (BM). A3: EDTRS grid delineating nine macular sectors. SMT, CMT and IMT were measured within the superior inner macula, the foveal and the inferior inner macula sector, respectively. B: IRLT and ORLT were assessed in the central macular region with 1000µm distance temporal to the fovea center and perpendicular to the retinal pigment epithelium (RPE). IRLT was measured from the ILM to the outer border of the outer plexiform layer (211 µm). ORLT was measured between the inner border of the outer nuclear layer and the inner border of the RPE (129 µm). CMT, central macular thickness; IMT, inferior macular thickness; IRLT, inner retinal layer thickness; ORLT, outer retinal layer thickness; SMT, superior macular thickness.


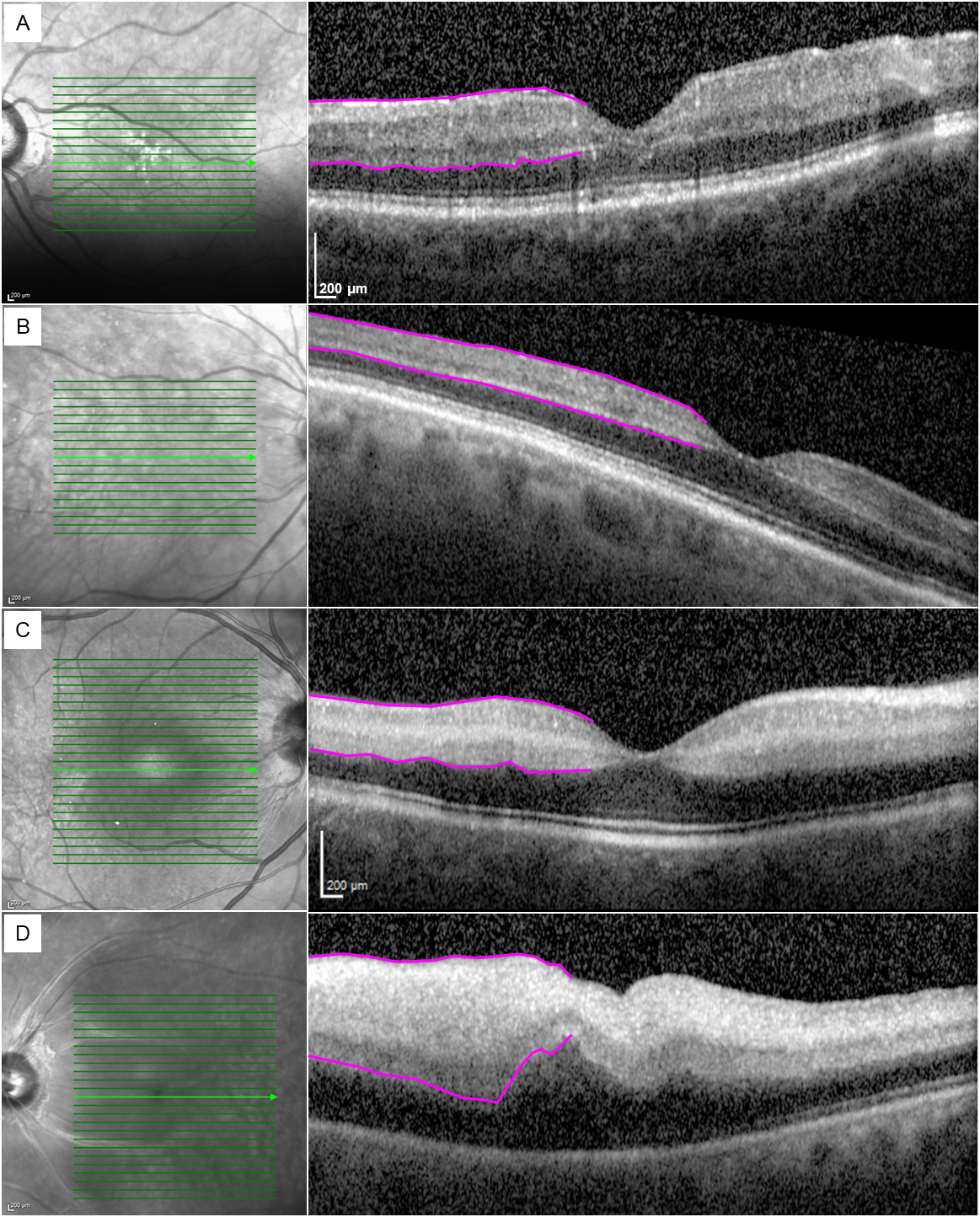


**Figure 2:** **Spectral-domain optical coherence tomography images of the macular and foveolar region (left row) depicting varying grades of inner retinal layer (IRL) hyperreflectivity in central retinal artery occlusion (CRAO).** Two-dimensional cross-section of the corresponding retinal layers (right row) with purple bordering indicating the IRL. A: Regular reflectivity of the IRL. Mild (B), moderate (C) and severe (D) hyperreflectivity of the macular IRL in patients with CRAO.


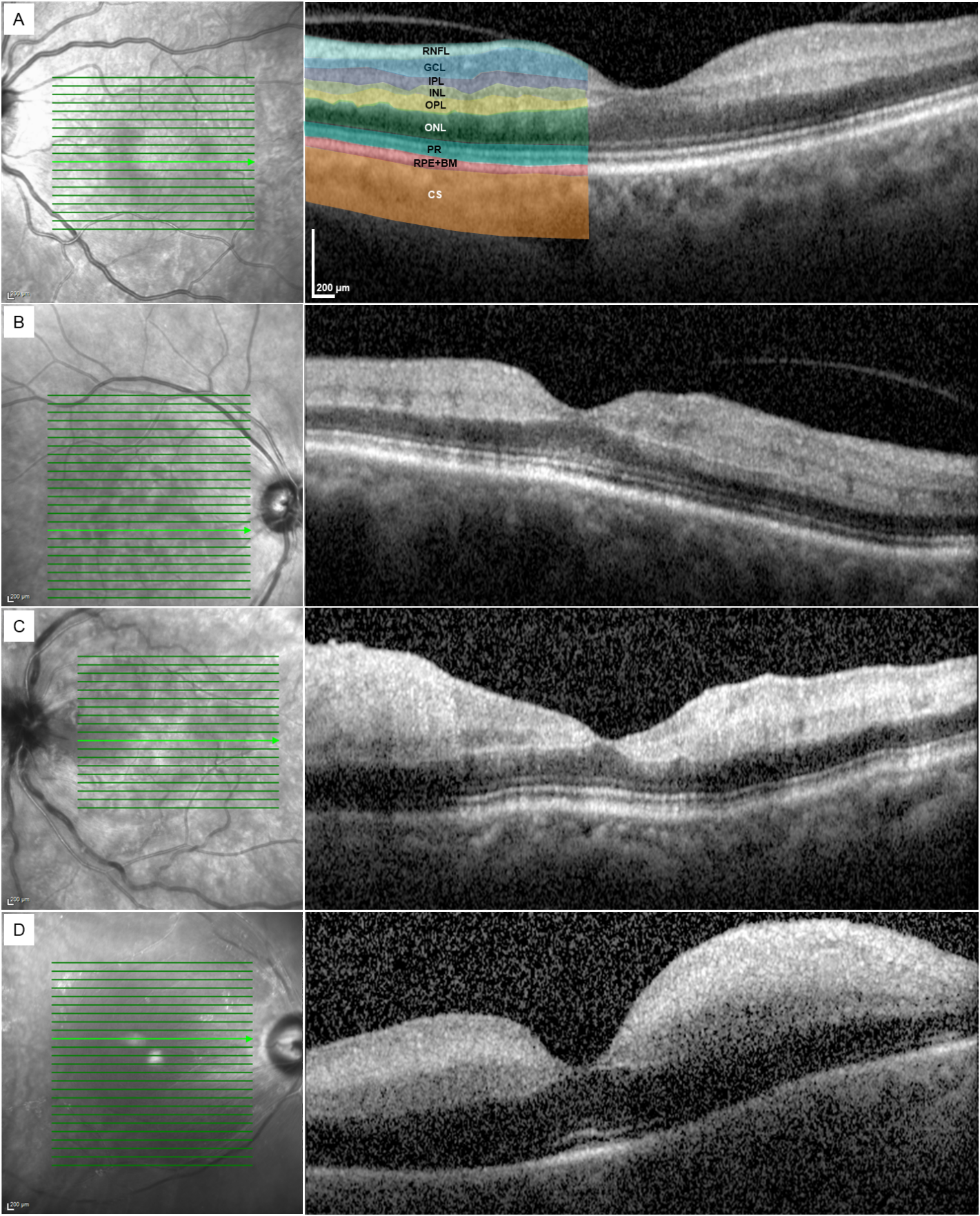


**Figure 3:** **Annotated spectral-domain optical coherence tomography images of the macular and foveolar region (left row) exemplifying varying grades of retinal layer structure loss in central retinal artery occlusion (right row).** A: Intact retinal layer structure as indicated by color overlay. B: Partial loss of retinal layer structure (grade I) with largely preserved retinal layering. B: Substantial loss of retinal layer structure (grade II) with some areas of preserved retinal layering. C: Complete loss of inner retinal layering (grade III). BM, Bruch’s membrane; CS, choroidal stroma; GCL, ganglion cell layer; INL, inner nuclear layer; IPL, inner plexiform layer; ONL, outer nuclear layer; OPL, outer plexiform layer; PR, photoreceptor layers; RNFL, retinal nerve fiber layer; RPE, retinal pigment epithelium.


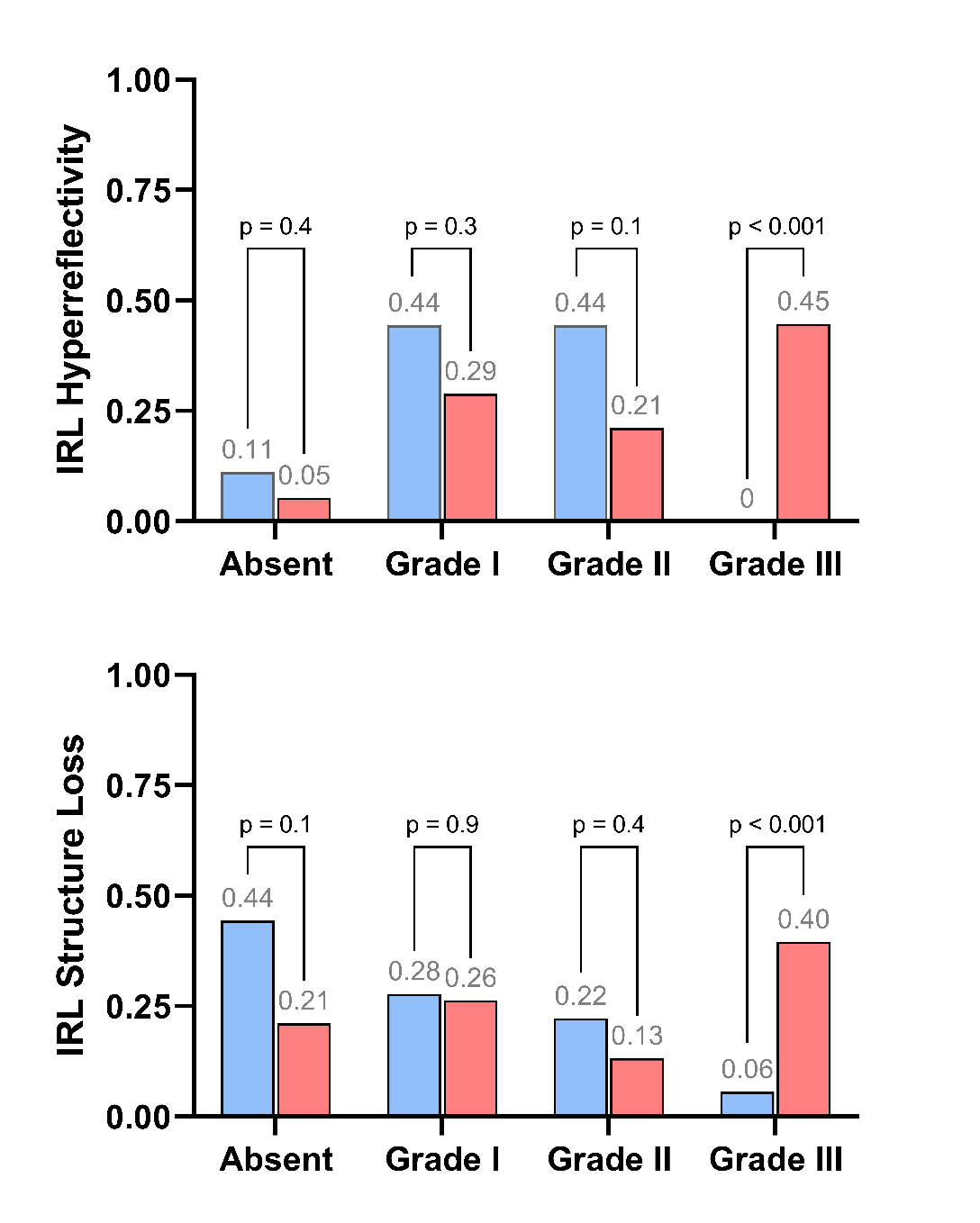


**Figure 4:** **Percentage distribution of inner retinal layer (IRL) hyperreflectivity and loss of IRL structure grading in CRAO patients with (red columns) and without (blue columns) visible RDR on DWI.** Increased frequency of grade III IRL hyperreflectivity (p = 0.0007) and grade III IRL structure loss (p = 0.009) was noted in patients with visible RDR on DWI. CRAO, central retinal artery occlusion; RDR, retinal diffusion-restriction.

**Literature**

1. Danyel LA, Miszczuk M, Connolly F, Villringer K, Bohner G, Rossel-Zemkouo M, et al. Time Course and Clinical Correlates of Retinal Diffusion Restrictions in Acute Central Retinal Artery Occlusion. AJNR Am J Neuroradiol. 2021.

2. Early Treatment Diabetic Retinopathy Study design and baseline patient characteristics. ETDRS report number 7. Ophthalmology. 1991;98(5 Suppl):741-56.
